# Hallux Osteoid Osteoma: A Case Report and Literature Review

**DOI:** 10.2174/1874325001711011066

**Published:** 2017-09-30

**Authors:** Konstantinos C. Xarchas, George Kyriakopoulos, Spyros Manthas, Leon Oikonomou

**Affiliations:** 1^st^ Department of Orthopaedics and Trauma, Athens General Hospital, G. Gennimatas, Athens, Greece

**Keywords:** Osteoid osteoma, Hallux, Foot, Tumours, Benign, Treatment

## Abstract

Osteoid osteoma is a benign bone tumour that mostly affects males in the second and third decade of their life. The lesion mainly occurs in long bones, usually in the femur and tibia, causing severe localized pain that is worse at night and responds to nonsteroidal anti-inflammatory drugs (NSAIDs). Diagnosis is usually made on the basis of history and radiographic findings. However, in more unusual locations as the hand and foot, diagnostic issues can arise. Treatment often includes complete removal of the tumor. We present a 22 year old male with osteoid osteoma involving the distal phalanx of the hallux. To our knowledge very few cases of great toe osteoid osteoma have been reported in the literature.

## 1 INTRODUCTION

Osteoid osteoma is a benign osteoblastic bone tumor, originally described in 1935 by Jaffe [1]. It is characterized by a well demarcated core of usually less than 1 cm, and by a distinctive surrounding zone of reactive bone formation [2].

Males in the second and third decades of life are most commonly affected [3], with findings of severe pain, typically worse at night and dramatically relieved by NSAIDs [4, 5].

Osteoid osteomas comprise approximately 10% of benign bone tumours [6] with almost 50% of cases involving the femur or the tibia [7]. Involvement of the hand and foot is rare, with phalangeal lesions of the foot amounting to 2% of all lesions [8].

Soft tissue swelling and nidus location in the cancellous or subperiosteal region, are unique features of finger and toe lesions [4, 9], that can mimic other conditions such as osteomyelitis and other bone tumors, therefore posing a diagnostic challenge.

Although the exact mechanism that leads to pain is not known, increased prostaglandin production and nerve fiber density in the nidus have been implicated [23-25]. A 100 to 1000fold increase in prostaglandin concentrations (PGE2) in the nidus compared to normal bone has been observed, and immunohistochemical staining demonstrated very high concentrations of COX-1 and COX-2, thus suggesting a mechanism for the response to NSAID analgesia. [26]

The characteristic pain pattern and clinical findings as swelling and deformity should raise a high suspicion for the diagnosis of osteoid osteoma (OO). Plain radiography is the initial diagnostic modality that can reveal the typical radiolucent nidus surrounded by an area of sclerotic bone. However, in more complex anatomic locations, bone scintigraphy has been used, where the lesion presents as a focal area of high uptake. Also, computerized tomography can provide better detail and aid in differential diagnosis. The use of MRI is somewhat controversial as it can obscure the differential diagnosis with more malignant lesions and lead to overtreatment.

Treatment of OO typically consists of a trial of medical management with NSAIDs with a dramatic decrease in symptoms, which can lead to resolution of the disease [27], although the long term effects and recurrence rates have not been clearly documented. Failure or intolerance of the medical management leads to surgical treatment, which mainly consists of open surgical resection and percutaneous radiofrequency ablation (RFA). Open surgical resection of the OO has been the golden standard in treatment, with minimal recurrence and resolution of symptoms. The morbidity and possible complexity of the open procedure together with the improvement in imaging modalities, have led to an increased use of percutaneous CT-guided techniques such as percutaneous RFA and percutaneous excision. Percutaneous excision involves the insertion of a curette over a CT guided placed wire in the nidus and excision of the lesion. The advantage of the method compared to RFA is the possibility of histologic diagnosis but cortical thinning may lead to a stress riser [28]. RFA has been shown to be a safe and effective treatment with low recurrence rates [29] and low morbidity. The main limitations of RFA are the lack of tissue for a histologic diagnosis, the possibility for damage to neural tissue if applied in close proximity and also skin damage.

## 2 CASE REPORT

A 22 year old male patient was admitted in our department with right hallux pain for the past 18 months. There was no history of injury. The pain was progressive and worse at night. The pain was initially relieved by NSAIDs but in the last few months only partially and the patient was forced to limit activities due to the pain. There were no constitutional symptoms such as fever, weight loss or malaise.

**Fig. (1):**
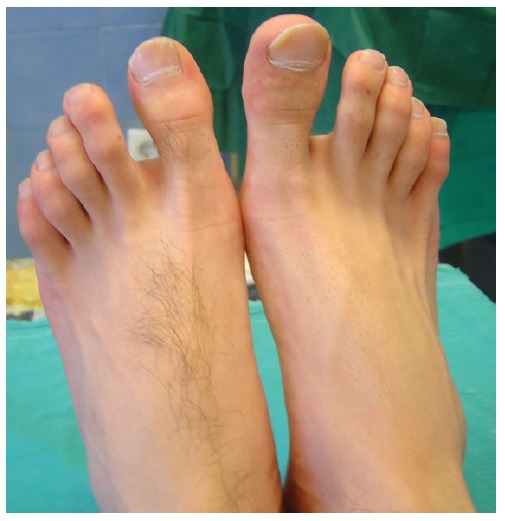
Swollen Right hallux compared to normal Left.

**Fig. (2):**
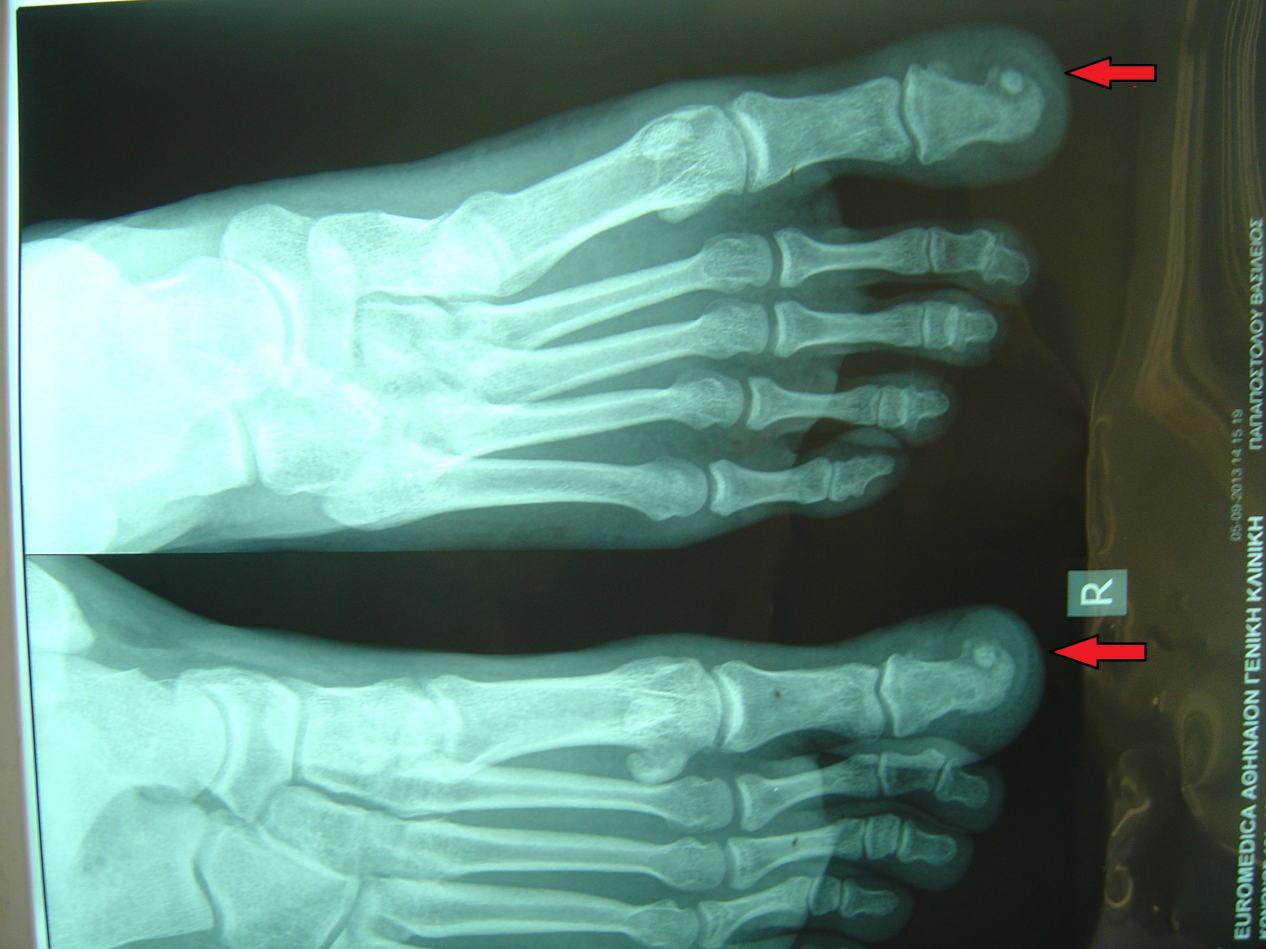
Xrays of the tumour AP and Lateral.

The distal phalanx of the right hallux was swollen and tender to palpation, with no obvious erythema or increase in temperature (Fig. **1**). The patient was otherwise fit and well. Blood analysis was unremarkable. Radiographic workup included plain x-rays of the foot and a CT scan and revealed a small radiolucency with cortical sclerosis and a nidus formation in the medial side of the distal phalanx of the great toe (Fig. **2**).

**Fig. (3):**
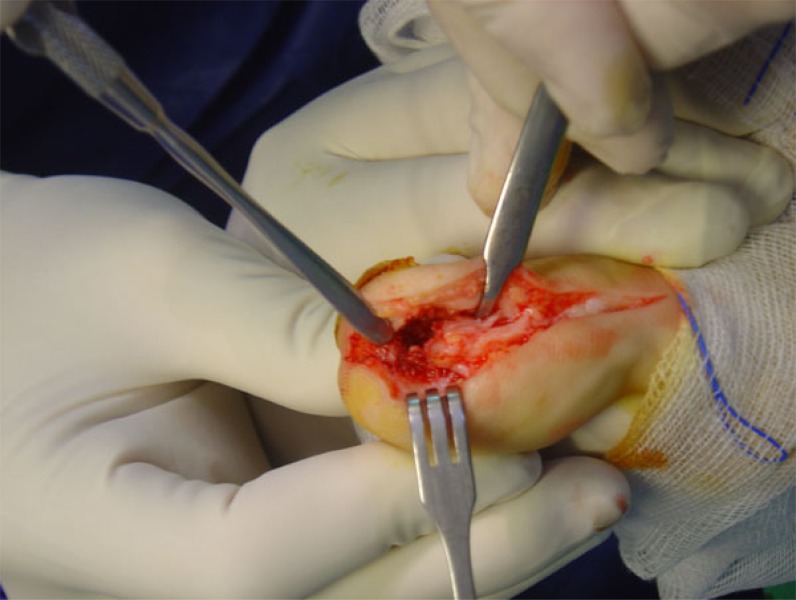
Surgical approach to the tumour.

**Fig. (4):**
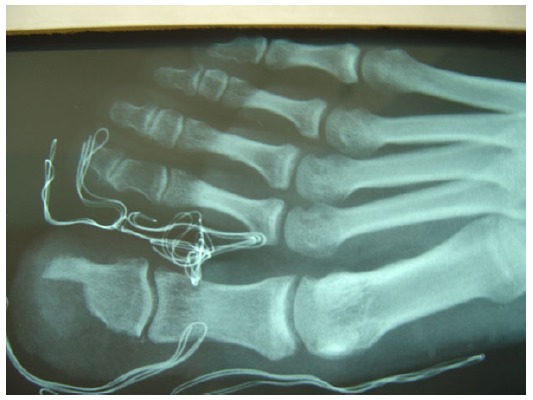
Postoperative Xray AP.

**Fig. (5):**
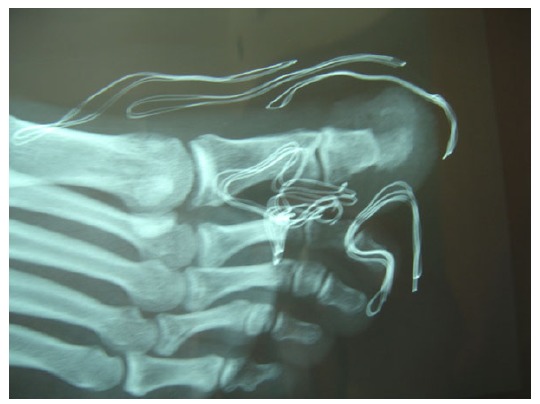
Postoperative Xray Lateral.

The patient underwent operative excision of the mass through an antero-medial incision with atraumatic elevation of the nail bed and nail in one layer (Fig. **3**). Excision of the nidus and surrounding bone to macroscopically healthy borders was performed and histologic samples were sent for analysis (Figs. **4** and **5**). The skin was closed and the nail was used as a splint to preserve the preoperative hallux shape. Pathology confirmed the diagnosis of osteoid osteoma. The patient was immediately relieved from nocturnal pains and progressively completely relieved from hallux pain. Immediately postoperatively, the patient was mobilized with a wedge shoe. At two years follow up, there were no signs of recurrence or skin and nail problems and the patient was ambulating freely.

## 3 MATERIALS AND METHODS

A systematic review of the PubMed Database was conducted. Search results are listed below (Table **1**), as well as the keywords that were used. Abstracts of cases in English language were exclusively included for review. Abstracts were reviewed and full text articles were subsequently obtained. Thirteen cases of great toe osteoid osteoma were found in the literature (Table **2**), making it a very rare or poorly reported entity.

## 4 DISCUSSION

Pain in the great toe of the foot is attributed to a wide variety of diseases, with the commonest being trauma, malalignment and degenerative disease, as well as less common causes such as infections of the bone (osteomyelitis or cellulitis) and tumours, both benign and malignant. Differential diagnosis should take into account conditions like osteomyelitis due to pseudomonas (mostly in diabetics), epidermoid inclusion cysts, chondrosarcoma, osteoblastoma, subungual squamous cell carcinoma, intracortical osteosarcoma and osteoid osteoma [21]. Diagnosis is usually delayed (mean 16 moths after onset of symptoms with a range of 8-36 months), and this was the case in our patient, where the diagnosis was made at 18 months after the onset of symptoms. This can be attributed to the diagnostic challenge posed by OO in the Hallux and also the pain relief experienced by NSAIDs, which in our case delayed the patient from seeking medical attention.In the rare occurrence of OO diagnosis, treatment consists of either open surgical excision of the tumour or percutaneous procedures, such as surgical resection, radio frequency or laser ablation, and ethanol injection [22]. The proximity of the lesion to the skin, the high frequency of complications (bone osteonecrosis, skin, tissue and neuromuscular injury) and the inability of histopathologic confirmation related to these procedures [22], along with our lack of experience in percutaneous techniques led to the decision for open surgical excision in our case. This decision seems to be in accordance with the literature, as none of the cases found were treated with percutaneous techniques. As the literature on such a rare entity is comprised of few case reports, data on outcomes and function of the treated cases is virtually non-existent. Patient outcomes in cases with no recurrence are in our opinion related to the size of the lesion excised and the quality of tissue reconstruction post excision.

## CONCLUSION

Osteoid osteoma is a well described rare benign tumour that mostly affects long bones. Localization of the tumor on the great toe of the foot, as described in our case, comprises a rare medical entity, as only few cases have been described according to literature. This fact illustrates the difficulties associated with the early diagnosis and treatment of the lesion. A combination of thorough medical history, radiographic examination and clinical suspicion should lead the clinician to the diagnosis. In our case, total resection of the lesion resulted in complete remission of the patient’s symptoms and no signs of recurrence at two years follow up.

## Figures and Tables

**Table 1:** Literature review results.

**Search keywords**	**Results**
Osteoid Osteoma	3000
Foot	123250
Hallux	5692
Toe	12513
Osteoid osteoma toe	29
Osteoid toe	30
Osteoid osteoma hallux	14
Osteoid hallux	15
Osteoid osteoma foot	194
Osteoid foot	174

**Table 2:** Literature review results.

**Paper**	**Patient age**	**Time** **to treatment**	**Type of treatment**	**Disease free FU**
Adler *et al.* [10]	23	24 months	Excision	N/A
Alkalay *et al.* [11]	22	1 year	Total excision	1 year
Bordelon *et al.* [9]	14	1 year	En bloc excision	N/A
Ekmekci *et al.* [12]	29	N/A	Total excision	N/A
Mohsen *et al.* [13]	32	8 months	Excision	N/A
Hakan *et al.* [14]	34	17 months	Excision	63 months
Hamilos *et al.* [15]	37	N/A	En bloc excision	6 years
Hattori *et al.* [16]	22	N/A	Excision	N/A
Jowett *et al.* [17]	20	36 months	Excision IP fusion	N/A
Kahn *et al.* [18]	32	1 year	En bloc excision	7 months
Oztürk *et al.* [19]	9	2 years	Excision	N/A
Spinosa *et al.* [20]	29	1 year	Excision	1 year
Turkmen *et al.* [21]	23	1 year	Excision	10 months
Yamaga *et al.* [22]	N/A	10 months	Excision	N/A

## LIST OF ABBREVIATION

OO: = Osteoid Osteoma
